# Callous-Unemotional Traits Moderate the Relation Between Prenatal Testosterone (2D:4D) and Externalising Behaviours in Children

**DOI:** 10.1007/s10578-016-0690-z

**Published:** 2016-10-12

**Authors:** Alyson Blanchard, Luna C. Munoz Centifanti

**Affiliations:** 1 Department of Psychology, Nottingham Trent University, Burton Street, Nottingham, NG1 4BU UK; 2 School of Psychology, University of Liverpool, Bedford Street South, Liverpool, L69 7ZA UK

**Keywords:** Callous-unemotional traits, Externalising behaviours, Maternal stress, Prenatal testosterone, 2D:4D, Moderation

## Abstract

Children who exhibit callous-unemotional (CU) traits are identified as developing particularly severe forms of externalising behaviours (EB). A number of risk factors have been identified in the development of CU traits, including biological, physiological, and genetic factors. However, prenatal testosterone (PT) remains un-investigated, yet could signal fetal programming of a combination of CU/EB. Using the 2D:4D digit ratio, the current study examined whether CU traits moderated the relationship between PT and EB. Hand scans were obtained from 79 children aged between 5 and 6 years old whose parents completed the parent report ICU (Inventory of Callous Unemotional Traits) and SDQ (Strengths and Difficulties Questionnaire). CU traits were found to moderate the relationship between PT and EB so that children who were exposed to increased PT and were higher in CU traits exhibited more EB. Findings emphasize the importance of recognising that vulnerability for EB that is accompanied by callousness may arise before birth.

## Introduction

Biological factors identify children with a profile of externalizing behaviours (EB) accompanied by callous-unemotional (CU) personality traits: lack of empathy, callous disregard for others’ wellbeing and their feelings, and a lack of responsibility and care over performance [1–3]. Biological factors may include exposure to hormones in the womb, which might set the stage for callous or cruelly perpetrated problem behaviors [4–6]. Given the relation between testosterone and psychopathy and our recent demonstration of an association between prenatal testosterone (PT; measured by the 2D:4D ratio) and primary and secondary psychopathy (callousness and the affective traits; antisocial and impulsive behaviours) [7], we investigate the possibility that exposure to PT is related to CU traits and EB. Research demonstrates that children with CU traits and EB evince biological profiles that are distinct from those children with CU traits alone or those with EB that are not accompanied by CU traits [2, 8]. Thus, we tested interactive effects of PT and CU traits on EB.

CU traits have been shown to differentiate distinct subgroups of children and adolescents with serious EB (e.g., conduct problems, conduct disorder, aggression and antisocial behaviour) [9]. For example, children with EB *and* CU traits have been characterized as temperamentally fearless with diminished emotionality, which is suggested to explain their propensity toward lifelong antisocial behavior [10, 11]. Children with CU traits and EB have reduced emotional and physiological reactivity that is not evident in children with EB alone [12, 13]. Their emotional deficits may be driven by a dysfunctional amygdala, given research showing reduced potentiated startle to violent images for those children with CU traits and EB [14]. Atypical neurological development may explain the hypoactivity to others’ distress, which impairs important brain regions for social and affective functioning [15–17]. Further, EB with CU traits show substantial heritability, greater than the heritability estimates for EB without CU traits [18]. These biological influences suggest that a lack of emotional and physiological reactivity to fearful events could explain why children with CU traits are less receptive to learning as a result of punitive measures, hindering normative social development, and predisposing these children to lifelong antisocial behavior [19].

Although the psychophysiological and biological profile of youths with CU traits is relatively well researched [20–22] the prenatal biology of children who later show CU traits is unknown. There is reason to speculate that PT exposure might play a role in the development of behaviours associated with psychopathy [23, 24]. For example, using the 2D:4D digit ratio as a biomarker for PT, research shows that high PT exposure is related to higher trait aggression [25], indirect and reactive aggression [26–28] sensation seeking and boredom susceptibility [29], recreational, financial and social risk taking [30, 31], increased sensitivity to status cues [32] and dis-inhibition [33]. In contrast, higher levels of prenatal estrogen (PE) are associated with empathy [34, 35], pro-social behaviour [36], neuroticism [29] and anxiety [37]. Thus, the masculinisation of the brain before birth appears to have long-lasting consequences for psychopathic behaviours, starting from childhood into adulthood.

One causal factor implicated in the fluctuation of PT is maternal stress, which could provide a proximate environmental determinant for the development of psychopathic behaviours. Maternal stress is hypothesised to elevate levels of cortisol, which, via the hypothalamic-pituitary adrenal (HPA) axis, act on the adrenal, ovarian/testicular functioning of the fetus thereby stimulating the production of PT [38–41]. Indeed, EB (e.g., aggression and sensation seeking) associated with higher levels of PT are also related to maternal anxiety [42, 43]. This may indicate a kind of “fetal programming”[44] whereby maternal stress acts as a cue that the environment outside the womb is stressful. Therefore, in-utero hormone levels may prompt masculinisation of the unborn infant’s brain to prepare them for a competitive environment. The child is then equipped with masculinsed traits and behaviours that are adaptive in the harsh environment that they are born into. However, studies also show that the 2D:4D ratio is moderately to highly heritable [45] and therefore, while PT is evidently an important contributor to the development of certain behaviours, both non-shared environmental factors and genetic influence should be taken into consideration.

Nevertheless, the relationship between testosterone and psychopathic behaviours is not entirely clear. For example, testosterone has been associated with impulsivity, and people with high testosterone readily activate aggressive coping strategies when provoked [46]. People with CU traits tend to show instrumental or planned aggressive behaviour rather than reactive or provoked aggression [47–49]. However, ratios between testosterone and cortisol, specifically lower ratios (i.e., lower levels of testosterone to higher levels of cortisol), have been argued to be indicative of good, rather than abnormal amygdala functioning, which is characteristic of CU traits and primary psychopathy [50]. As expected, people with high ratios, indicating high levels of testosterone and low levels of cortisol, have been shown to be high on psychopathic traits [51, 52]. Interestingly, a similar finding has emerged from a 2D:4D ratio study of adolescent males in which those that had been exposed to higher levels of PT, low cortisol reactivity was associated with self-reported aggression and rule-breaking behaviour [53]. Thus, the relation between testosterone and CU traits may be complex and involves interacting hormonal systems.

In light of the current literature, we examined whether children between the ages of 5 and 6 years who were exposed to greater levels of PT expressed higher levels of CU traits and EB. Children at the age of 5–6 years are the age at which they enter school, and this group is of particular relevance to study because of developments in empathy, emotion understanding, and cognition that demonstrate extensive growth at this age [54]. Additionally, empathy and emotion understanding deficits have been found to be associated with CU traits at this age [55]. Children who are entering school are in a position to develop independence from their parents and therefore become susceptible to positive and negative peer influences [56, 57]. Furthermore, CU traits have yet to be examined in this particular age group. Previous studies have shown that CU traits emerge as early as age 2 years [58, 59] and remain relatively stable throughout childhood [59–61]. Therefore, the influence of prenatal experiences in the development of CU traits and EB may be observable in our sample of children. Studies investigating “fetal programming” [7, 62] with regards to psychopathic and antisocial behaviour have also so far only concerned adults.

Therefore, based on our prior research [7] and that both genes and environmental (i.e., maternal stress) factors are implicated in the status of PT, we expected elevated levels of PT to be associated with higher CU traits and higher EB. Considering that the presence of CU traits combined with EB designates a unique group of children with serious EB, we hypothesized an interaction between exposure to increased PT and more CU traits in the expression of higher levels of EB.

## Method

### Participants and Procedure

Seventy-nine parents and children (48 girls) were recruited from five primary schools in the Merseyside area of the United Kingdom. Schools came from areas of varying socio-economic backgrounds as indicated by their Index of Multiple Deprivation (IMD) score, ranging from 3.40 to 47.93. Four children came from 10 % of the most deprived areas; 24 children came from the 20 % most deprived areas; nine children came from the 40 % most deprived areas and 43 children came from the 10 % least deprived areas in England. Children were in Year 1 of The British Education System and aged between 5 and 6 years.

### Measures

#### Inventory of Callous-Unemotional Traits (ICU)

The ICU Parent Report [63] is a 24-item questionnaire that assesses CU traits in children. Using a four-point Likert scale, the parent rates how true (*0* = not at all true, *3* = definitely true) certain statements are of their child (e.g., “Does not show emotions” and “Does not care about doing things well”). Ratings are summed to produce an overall score of CU traits. Internal consistency was good and was improved by removing item 10 (“Does not let feelings control him/her”) (Cronbach’s alpha = 0.83), which is a consistent underperformer in prior research [64].

#### Strengths and Difficulties Questionnaire (SDQ)

The SDQ [65] is a 25-item questionnaire that screens for various positive and negative behaviours. Each subscale consists of five items, and we combined the Conduct Problems and Hyperactivity subscales to produce an overall Externalising score, as has been done in prior research [55]. On a three-point Likert scale, parents rated how true (*0* = not true, *2* = certainly true) statements such as: “Often lies or cheats” (Conduct problems), and “Restless, overactive” (Hyperactivity) were of their child. The Externalising score produced acceptable internal consistency (Cronbach’s alpha = 0.76).

#### Prenatal Testosterone Exposure

The 2D:4D digit ratio is an accepted measure for PT exposure [66–68] and is calculated by dividing the length of the second finger digit (2D) by the length of the fourth finger digit (4D). Sexual dimorphism in 2D:4D is present from at least the 14th week of pregnancy and remains stable into adulthood [69–73]. Postnatal hormonal surges also drive finger length growth; however research shows that high levels of circulating testosterone during adolescence actually reduce the impact of stress [74]. Therefore, the 2D:4D ratio should be robust to postnatal stresses.

We used a Canon Canoscan LiDE120 scanner to obtain hand scans from which fingers were measured using the measurement tool in Adobe Photoshop CS5. Hand scans and computer-assisted measurement are argued as a preferable method to using callipers or rulers [75, 76]. The length of the finger measurement is taken from the tip of the finger to the proximal crease of the palm. Both right-hand (RH) and left-hand (LH) ratios were calculated. Inter-observer repeatabilities of the finger measurements were assessed using Intraclass correlation coefficients (ICCs) [77] and revealed good reliability between two observers. ICCs were 0.848 for R2D, 0.868 for R4D, 0.347 for RH2D4D, 0.892 for L2D, 0.913 for L4D and 0.468 for LH2D:4D (all *ps* < 0.001).

### Procedure

Head Teachers were approached via email or telephone and were provided with an Access Letter that described the nature and purpose of the study, and the data collection process. On obtaining authorization for the study to be carried out, individual study packs for each child containing an Information Sheet, Consent Forms (Parent Consent for child participation, Child Consent and Parent Consent), ICU and SDQ were sent to the school. The Information Sheet stated the nature and purpose of the study; that it involved the parent completing two questionnaires about their child’s behaviour, and for their child’s hands to be scanned at school. Teacherd distributed the packs to children who were to take them home to their parents. A period of at least two weeks was given for parents to return the packs (in a sealed envelope provided) with completed consent forms and questionnaires. The children whose parents had consented for them to take part were asked for their consent. If they agreed, they had their hand scanned at a later date whilst they were at school.

## Results

**Fig. 1:**
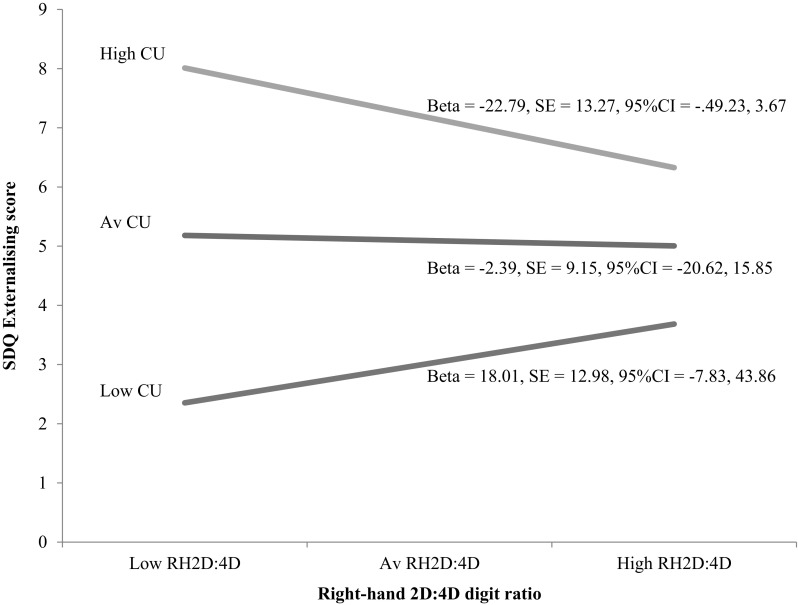
Externalising scores for exposure to prenatal testosterone (RH2D:4D), split by *low, average* and *high CU* traits scores

Descriptive statistics are shown in Table 1. Digit ratios for both hands were smaller in boys than in girls but were not significantly so. Boys also scored higher in all reported measures, but not significantly. In order to look at the relationship between 2D:4D ratio, CU traits and EB, we conducted a series of zero-order correlations (Table 2). Due to multiple comparisons and the increased likelihood of making a Type 1 error, a Bonferonni correction set the minimum alpha level to 0.001 [78]. No significant relationships at 0.001 were found between any of the variables. Next we conducted two stepwise regression analyses (Table 3) where in the first step either RH or LH2D:4D and CU traits were regressed onto the SDQ Externalising score, and then on the second step we added an interaction term of either RH × CU traits or LH2D:4D × CU traits. In the first step of the RH2D:4D model, SDQ Externalising was significantly and uniquely predicted by CU traits, but not RH2D:4D. With the addition of the interaction term, CU remained a significant predictor, and the standardised beta for RH2D:4D became significant. The interaction term was also significant. The interaction between RH2D:4D and CU traits explained 4 % of the variance in SDQ Externalising. In the first step of the LH2D:4D model, SDQ Externalising was uniquely predicted by CU traits. Neither LH2D:4D nor the interaction term was significant in predicting SDQ Externalising scores. Post hoc testing was applied using PROCESS [79] to examine the association between RH2D:4D and SDQ Externalising at low (−1SD), mean, and high (+1SD) levels of CU traits. The form of the interaction is shown in Fig. 1.

**Table 1:** Means and stand deviations for all variables

	Total	Boys	Girls	*t*
RH2D:4D	0.956 (0.037)	0.956 (0.036)	0.956 (0.037)	−0.09
LH2D:4D	0.963 (0.038)	0.959 (0.037)	0.965 (0.039)	−0.72
ICU	16.34 (4.64)	17.26 (5.26)	15.83 (4.15)	1.34
SDQ Externalising	4.92 (3.47)	5.48 (3.54)	4.56 (3.41)	1.15

Comparisons are between boys and girls

**Table 2:** Zero order correlations for RH2D:4D and LH2D:4D

	RH2D:4D	LH2D:4D	ICU	SDQ externalising
RH2D:4D	1	0.74***	0.23*	0.10
LH2D:4D		1	0.14	0.11
ICU			1	0.47***
SDQ externalising				1

*p < .05

**p < .01

***p < .001

**Table 3:** Stepwise regression of 2D:4D and CU traits on SDQ Externalising scores

	SDQ (RH2D:4D)				SDQ (LH2D:4D)			
	B	SE	*β*	∆R²	B	SE	*β*	∆R²
Step 1								
2D:4D	−1.93	9.37	−0.02		2.95	9	0.03	
CU traits	0.04	0.08	0.54**	15.12**	0.40	0.07	0.53**	15.17**
Step 2								
2D:4D	69.7	34.28	0.74*		39.61	32.45	0.43	
CU traits	4.65	1.96	6.21		2.44	1.74	3.26	
2D:4D × CU traits	−4.34	2.03	−5.9	4.70*	−2.12	1.81	−2.82	1.38

SDQ and RH2D:4D model: *R* ^*2*^ = 0.33, *F* (3, 78) = 12.14, *p* < .001; Step 1: ∆R² = 0.29, *F* (2, 76) = 15.12, *p* < .001; Step 2: ∆R² = 0.04, *F* (1, 75) = 4.70, *p* = .03. SDQ and LH2D:4D model: *R* ^2^ = 0.30, *F* (3, 78) = 10.62, *p* < .001; Step 1: ∆R² = 0.29, *F* (2, 76) = 15.12, *p* < .001; Step 2: ∆R² = 0.01, *F* (1, 75) = 1.38, *p* = ns

*p < .05

**p < .01

## Discussion

We investigated whether PT was related to CU traits and EB in children between 5 and 6 years of age. We also examined whether there was an interaction between exposure to PT and CU traits in being associated with higher levels of EB. Indeed, we found that children who were higher in CU traits and who had been exposed to greater levels of PT were higher in EB reported by parents. Children who were higher in CU traits, but who had been exposed to lower levels of PT (i.e., indicative of greater prenatal estrogen), demonstrated fewer EB. This finding suggests that CU traits can worsen or enhance the masculinising influence of PT in the development of EB. To our knowledge, this is the first study to suggest that prenatal neuroendocrinology may be a factor involved in CU traits and EB exhibited in children aged between 5 and 6 years old.

Our findings are consistent with what was understood about the interplay between genetic and environmental factors in the development of child psychopathy. Although our study is the first to demonstrate that this interplay may start before birth, research reliably indicates that some children are genetically vulnerable to the development of a cold and callous temperament style of interacting with others. Such findings are demonstrated in 7-year olds [20, 80, 81], 9–10 year olds [82], 12-year olds [83], adolescents [84, 85] and adults [86]. However, children with CU traits may be further exposed to stressors that result in pervasive and serious EB because of how they interact with their environment. For example, children with CU traits have been shown to experience greater negative life events over time, which may be a consequence of their own fearless and risky behaviours that lead to encountering dangerous environments or situations where they are likely to suffer stressful events [87–91]. Our main finding could suggest that a child with a genetic vulnerability to CU traits is already, *pre-birth*, susceptible to developing EB caused by environmental factors; in this case, elevated levels of PT activated by maternal stress. Specifically, the high-CU child may be more sensitive to the masculinising effects that higher levels of PT have on neural organisation. We would like to address whether it is adaptive to be predisposed to problem behaviours so early on in life. Essentially, does our finding indicate a potential role for fetal programming [44]?

Maternal stress has been suggested to operate as a signal of impending harsh environmental conditions to the fetus. Specifically, stress increases cortisol, which changes fetal adrenal, ovarian and testicular functioning, and therefore PT production [38–41]. We would therefore expect an association between higher levels of PT and aggressive, competitive behaviours once entering the world. Fetal programming predicts that this association should be observed in both adults *and* children because an early start to problem behaviour would be adaptive in successfully navigating a hostile environment through to adulthood. Yet, a range of diverse and unaccounted for factors may act on PT and thus the association may not be straightforward. For example, PT levels are highly heritable [45] and thus genetic effect should be given due consideration. In which case, one might observe externalizing behaviour in those high on CU traits and PT regardless of the harshness of the environment. Thus, when either researching or in treatment planning, the child’s entire life history, including whether the mother experienced stress during pregnancy, is needed to construct the most informative account of their developmental trajectory to problem behaviour. This might also be helpful in prevention, by monitoring the expectant mothers mental health and intervening as appropriate (e.g., additional support) during pregnancy.

Our results suggest that from pre-birth, children with CU traits who were also exposed to more PT, are potentially more liable in developing behaviours that are adaptive in harsh environments, thereby providing some support for “fetal programming”. Interestingly, our findings corroborate what prior studies revealed. Namely, that high PT is related to hyperactivity, ADHD symptoms, conduct problems and poor social cognitive functioning in children from 3 to 7 years of age [36, 37]. We extend these findings to include children who exhibit traits and behaviours associated with child psychopathy.

The case for fetal programming is therefore gaining support, although further investigation is needed to identify the precise biological mechanism between maternal stress and PT, which currently remains a topic for investigation. The fetal programming hypothesis is challenged by high heritability values for 2D:4D [45, 92], and therefore multiple factors (biological *and* environmental) need consideration. Perhaps there is an association between the genes that code for CU traits and those that code for PT levels. However, in our study, we did not find evidence for zero-order correlations between CU traits and PT. It should be borne in mind that phenotypic output is the product of a highly complex process involving genes, the environment and gene x environment interactions. Therefore we can only speculate as to the implications of our results at this time.

A final point of interest is that children exposed to higher levels of PE had fewer EB only if they were higher on CU traits. Research reliably shows that high PE is associated with empathy and prosocial (need ref) behaviour [5, 34, 35], as well as anxiety and neuroticism [37, 93, 94]. Seeing that psychopathy overall is hypothesised to be a male adaptation [95], it is possible to speculate that feminising effects of PE counterbalance CU traits by some yet unknown mechanism. Children high on CU traits and exposed to higher levels of PE may not end up eliciting adverse reactions from parents or peers, perhaps because they are more prosocial or empathetic, at least cognitively rather than affectively (i.e., they can “talk the talk”). Consequently, they reduce the likelihood of developing EB usually associated with harsh environments. Of particular relevance is evidence from prior 2D:4D ratio research where PT moderated the association between exposure to aggression cues and prosocial behaviour. Specifically, individuals exposed to higher levels of PE became more prosocial in the presence of an aggression cue [32]. The authors suggested that contextual cues should be considered as moderating effects when interpreting associations between PT and personality traits, and might explain why findings from 2D:4D research can produce inconsistent results [32]. Our findings similarly highlight the need to consider other factors that might potentially moderate the relationship between PT and personality.

There are limitations to our study. We used the parent report versions of both the SDQ and ICU, which increases the potential for shared-method variance. It would have been beneficial to include the teacher report versions by way of verification. However, due to the need to limit the time required by the school to administer the data collection, we felt that the parent report versions were adequate. Assigning CU traits as the main focus for psychopathy research in children has also been challenged. Some argue that this ignores other important behavioural and interpersonal aspects of psychopathic personality that the ICU does not measure [96]. However, research consistently demonstrates CU traits as a key factor in identifying children who go on to develop psychopathy [9–11]. In the absence of equally compelling research that pinpoints another key factor, we are confident in using the ICU as an indicator of child psychopathic behaviour. Debate also surrounds the accuracy of using the 2D:4D ratio as a biomarker for PT and results can be inconsistent across sex, and even studies investigating similar behaviours [97]. However, there is good evidence to suggest that the 2D:4D reflects PT exposure [66–68] and has become a popular measure used in studies over the last 15 years. We therefore feel that our results make a unique contribution to the literature and provide the basis for investigating the subject area further. A final issue concerns sex differences. Both psychopathy and 2D:4D ratio are sexually dimorphic, however in our study, due to our sample size, to compare boys and girls would have failed to generate enough power to make any findings truly inferential. Future studies should aim to investigate gender as a potential moderator.

Our study is unique in including children. Studies that have linked aggression [25–27, 98]; although c.f. [99], sensation seeking [29], low empathy [4, 5, 34, 35], dominance [100] and antisocial behaviour [24] to high PT have only used adult samples, and there are almost no studies in children. Our findings will also contribute to the further advancement of developmental psychopathological theories of CP behaviour. Frick and colleagues [9] state that the most sufficient causal model can only be achieved by considering multiple risk factors, both biological and environmental. We have not only presented a hitherto unconsidered risk factor, but have also highlighted the timing for when this risk factor (e.g., before birth) takes effect. The most comprehensive theory helps to improve prevention or intervention treatment for at-risk or affected children. Indeed, early intervention is more effective [101–104] and may prevent the negative consequences of harsh parenting [57, 105]. Pregnant women who are encountering stress should be identified early in the pregnancy so that they can be provided with mental health support. Unresolved maternal stress may also make these women more vulnerable to developing a harsh parenting style; thus early intervention is critical to prevention of CP [32, 96].

Our findings contribute to an ever increasing and important body of research in child psychopathy. Researchers acknowledge that developmental pathways to adult psychopathy are not easily discovered and concern varied environmental and biological factors [9]. This is the first study to bring forward another biological factor in the form of PT, and therefore highlights the need to acknowledge that children are on the path to problem behaviour even before they are born.

## Summary

CU traits are readily acknowledged as the key to the development of serious EB behaviour in children. Multiple risk factors for CU combined with EB have already been identified in previous studies. However, this is the first to examine if prenatal experiences also contribute to this type of behaviour. Studies show that adverse traits and behaviours are expressed in adults who were subject to higher levels of PT, thereby highlighting PT as a potential risk factor for CU traits in children. Hence, in the current study, we examined CU traits as a moderating factor in the association between exposure to PT and EB. The 2D:4D digit ratio was used to measure exposure to PT in children 5–6 years old, who were also evaluated for CU traits and EB by their parents. A moderating effect was found for CU traits such that children exposed to higher levels of PT expressed more EB if they were high in CU traits. Conversely, children exposed to lower levels of PT but were high in CU traits expressed less EB. These findings suggest that CU traits can enhance or weaken the influence of prenatal masculinisation on CP EB. This study has therefore provided a fresh perspective on CU traits and EB in children by highlighting neuroendocrinoloy and prenatal experiences as potential factors in their development.
